# Afatinib in Non–Small Cell Lung Cancer

**Published:** 2015-09-01

**Authors:** Scott M. Wirth

**Affiliations:** University of Illinois Hospital and Health Sciences System and the University of Illinois at Chicago College of Pharmacy, Chicago, Illinois

Lung cancer is the second most common cancer and the leading cause of cancer-related deaths in both men and women (Siegel, Ma, Zou, & Jemal, 2014). Non–small cell lung cancer (NSCLC) is the most common form of lung cancer, accounting for about 85% of all lung cancers ((Molina, Yang, Cassivi, Schild, & Adjei, 2008). The treatment paradigm for NSCLC is rapidly evolving to incorporate specific treatment options considering both histology and molecular biomarkers for individual tumors ((Ettinger et al., 2014).

The emergence of targeted agents has been particularly important in improving care for patients with NSCLC, as multiple molecular biomarkers have been discovered to be important to tumor growth (Sequist et al., 2011; (Domvri et al., 2013). Currently, approved agents in the United States target tumors with anaplastic lymphoma kinase (*ALK*) gene rearrangements (ceritinib [Zykadia] and crizotinib [Xalkori]), vascular endothelial growth factor (VEGF) signaling (bevacizumab [Avastin] and ramucirumab [Cyramza]), programmed cell death 1 (PD-1) receptor signaling (nivolumab [Opdivo]), and epidermal growth factor receptor (EGFR) signaling (afatinib [Gilotrif], erlotinib [Tarceva], and gefitinib [Iressa]). Targeting EGFR has become of particular interest over the past decade due to its ability to activate multiple downstream growth pathways in solid tumors ((Domvri et al., 2013).

EGFR is part of a group of tyrosine kinase receptors also referred to as the HER or ErbB family ((Modjtahedi, Cho, Michel, & Solca, 2014). The family includes EGFR (HER1/ErbB1), HER2 (ErbB2), HER3 (ErbB3), and HER4 (ErbB4). *EGFR* mutations and HER2 overexpression have been shown to be prevalent in NSCLC tumors, particularly adenocarcinomas (Bonanno, Favaretto, Rugge, Taron, & Rosell, 2011). The most common mutations in *EGFR* include exon 19 deletion mutations and *L858R* (exon 21) substitution mutations ((Eberhard et al., 2005).

The first-generation reversible EGFR tyrosine kinase oral inhibitors erlotinib and gefitinib specifically target the EGFR receptor and have efficacy in patients with *EGFR* mutations (Fry, 2003). Erlotinib is readily available in the United States, whereas gefitinib is only indicated first line in combination with a US Food and Drug Administration (FDA)-approved test (Genentech, Inc., 2015; AstraZeneca, 2015). Although the first-generation agents have efficacy in *EGFR*-mutated NSCLC, resistance to these agents can occur most commonly through the acquisition of a secondary mutation such as *T790M*, which is found on exon 20 (Bonanno et al., 2011).

Afatinib, a second-generation irreversible ErbB family inhibitor, has been approved by the FDA for treatment of patients with *EGFR*-mutated NSCLC. Afatinib’s ability to irreversibly inhibit *EGFR* as well as other targets within the ErbB family may improve upon first-generation EGFR inhibitors and possibly overcome resistance to these agents.

## Pharmacology and Mechanism of Action

Afatinib is a second-generation anilinoquinazoline that irreversibly binds to an intracellular tyrosine kinase domain, subsequently inhibiting members of the ErbB receptor family (Li et al., 2008). Most specifically, afatinib inhibits EGFR (ErbB1), HER2 (ErbB2), and HER4 (ErbB4) receptors. The ability to inhibit multiple targets may be an advantage over erlotinib and gefitinib, which reversibly inhibit only EGFR (ErbB1; Fry, 2003). Afatinib’s irreversible binding properties may also be an advantage in inhibiting mutant cell lines, including *EGFR L858R/T790M* mutations, which are often resistant to erlotinib and gefitinib (Li et al., 2008; Kwak et al., 2005).

## Clinical Trials

Multiple phase I studies have been conducted in patients with solid tumors, including some with NSCLC (Agus, Terlizzi, Stopfer, Amelsberg, & Gordon, 2006; Yap et al., 2010; Eskens et al., 2008). In the phase I studies with continuous daily dosing (Agus et al., 2006; Yap et al., 2010), it was determined that the maximum tolerated dose (MTD) for afatinib is 40 to 50 mg orally once daily. Efficacy was suggested in one of these studies, in which four patients with NSCLC had a PR as determined by Response Evaluation Criteria in Solid Tumors (RECIST) criteria, two of which had *EGFR* exon 19 deletions (Yap et al., 2010).

**Second-Line Therapy**

A subsequent phase II, single-arm, open-label study (LUX-Lung 2) evaluating patients with NSCLC harboring *EGFR* mutations (exons 18 to 21) was conducted in 129 patients, 68 of whom received afatinib after first-line chemotherapy (Yang et al., 2012). Patients were excluded if they had previously received agents that inhibited EGFR. Afatinib was administered at a dose of 50 mg daily, which was later decreased to 40 mg daily after a protocol amendment due to tolerability.

An objective response was found in 57% of patients treated in the second-line setting, and this was not significantly different from that in treatment-naive patients (odds ratio [OR] = 0.71; 95% confidence interval [CI], 0.35–1.44). Median progression-free survival (PFS) in the entire population was 10.1 months (95% CI, 8.12–13.80), but it was shown to be longer in those with common *EGFR* mutations (exon 19 deletion, *L858R*) than in those with other uncommon mutations. Patients with common mutations also had a shorter PFS in the second-line setting vs. those in the first-line setting.

Afatinib has also been evaluated in patients with advanced NSCLC with previous exposure to EGFR inhibitors. In a phase IIb/III double-blind controlled trial of 585 patients (LUX-Lung 1), patients were randomized to receive afatinib 50 mg daily with best supportive care (BSC) vs. placebo with BSC (Miller et al., 2012). All patients had received previous chemotherapy and an EGFR tyrosine kinase inhibitor (erlotinib and/or gefitinib). *EGFR* mutation status was not required for study entry; however, those patients with known *EGFR* mutation status were included in the subgroup analysis. A post-hoc analysis was also performed for patients who were considered to have acquired resistance to previous EGFR tyrosine kinase inhibitor use. The primary endpoint of the trial was overall survival (OS), with secondary endpoints including PFS and objective response rate (ORR).

Upon trial completion, OS was not significantly different between those who received afatinib vs. those who received placebo (10.8 vs. 12.0 months; hazard ratio [HR] = 1.08; 95% CI. 0.86–1.35). However, PFS was improved in those receiving afatinib (3.3 vs. 1.1 months; HR = 0.38; 95% CI, 0.31–0.48), and confirmed ORR was also improved in those receiving afatinib per independent review (7% vs. < 1%, *p* = .0071).

When *EGFR* mutation status was evaluated, the PFS advantage for afatinib was significant for the 96 patients who were EGFR mutation-positive (3.3 months vs. 1.0 month; HR = 0.51; 95% CI. 0.31−0.85) but not for the 45 patients who were known *EGFR* mutation-negative. In contrast, OS was not significantly improved for afatinib in those who were *EGFR* mutation-positive. In those with known acquired resistance, PFS was also improved for those receiving afatinib vs. those who did not (4.53 months vs. 0.99 months; HR = 0.37; 95% CI, 0.26−0.52).

Furthermore, a similar phase II trial (LUX-Lung 4) evaluated the use of afatinib in patients who progressed on EGFR tyrosine kinase inhibitors (Katakami et al., 2013). All patients received afatinib at a starting dose of 50 mg daily. Median PFS was found to be 4.4 months by independent review. Those considered to have acquired resistance to previous EGFR tyrosine kinase inhibitors also had a median PFS of 4.4 months, similar to that found in the LUX-Lung 1 trial.

Results for second-line afatinib in squamous cell histology have also been recently reported, suggesting efficacy in patients with histologies other than adenocarcinoma (Soriaet al., 2015). In the LUX-Lung 8 trial, afatinib was directly compared with erlotinib following platinum-based doublet therapy (no prior EGFR tyrosine kinase inhibitor therapy was allowed). Results revealed an improved PFS and OS compared with erlotinib. Further comparative studies will be necessary to determine whether this is the preferred approach in this subset of patients, especially with the recent approval of nivolumab (Opdivo, single agent) and ramucirumab (in combination with chemotherapy). These agents have also shown to be efficacious in this setting, although they were compared with chemotherapy and not EGFR tyrosine kinase inhibitors (Garon et al. 2014, Brahmer et al., 2015).

**First-Line Therapy**

Two phase III randomized trials have been performed in the first-line setting for patients with advanced NSCLC and *EGFR* mutations (Sequist et al., 2013; Wu et al., 2014). Afatinib was approved following an open-label, randomized phase III study (LUX-Lung 3) in which it was compared with cisplatin and pemetrexed (Alimta) chemotherapy given every 21 days (Sequist et al., 2013). Patients were stratified based on race (Asian vs. non-Asian) and type of *EGFR* mutation (*L858R*, exon 19 deletions, or other mutation). The primary endpoint of the trial was PFS. Multiple secondary endpoints, including ORR, disease control rate, and OS, were also evaluated. Patients received afatinib at a dose of 40 mg daily, with the possibility to escalate to 50 mg daily after the first cycle if they did not experience any adverse events (such as rash, diarrhea, mucositis, or any other event greater than grade 1). A total of 345 patients were randomized to receive treatment, with a median follow-up of 16.4 months.

Therapy with afatinib resulted in a 4.2-month improvement in PFS compared with treatment with chemotherapy based on independent review (11.1 vs, 6.9 months; HR = 0.58; 95% CI, 0.43–0.78). Patients with common *EGFR* mutations (*L858R*/exon 19 deletion) received an even greater median PFS advantage (13.6 vs. 6.9 months; HR = 0.47; 95% CI, 0.34– 0.65).

Afatinib was also evaluated in an open-label, randomized phase III study (LUX-Lung 6) in which it was compared with the combination of cisplatin and gemcitabine chemotherapy (Wu et al., 2014). Afatinib was given at a dose of 40 mg daily. Similar to the previous study, patients were stratified by type of *EGFR* mutation. The primary endpoint of the trial was PFS.

After a median follow-up of 16.6 months, independent assessment of PFS was 11.0 months for afatinib, compared with 5.6 months for the combination of gemcitabine and cisplatin (HR = 0.28; 95% CI, 0.20–0.39), and a significant improvement was maintained across nearly all subgroups. Significant improvement in key secondary endpoints was observed for afatinib over chemotherapy in regard to ORR (66.9% vs. 23.0%; OR = 7.28; 95% CI, 4.36−12.18) and disease control rate (92.6% vs. 76.2%; OR = 3.84; 95% CI, 2.04−7.24). Three patients in this trial had *T790M* mutations, and one patient in each group had a PR.

At the time these results were reported, OS was immature in both phase III trials. However, a recent analysis of these trials indicates that OS was not significantly different between the afatinib and either of the chemotherapy groups (Yang et al., 2015). However, subgroup analysis revealed an increased survival for afatinib vs. chemotherapy in those with *EGFR* exon 19 deletion but not in those with *L858R* substitution mutations.

## Adverse Events

In the two phase III trials (LUX-Lung 3 and LUX-Lung 6) conducted in patients receiving afatinib for first-line therapy (Sequist et al., 2013; Wu et al., 2014), the most common adverse events (all grades) reported included diarrhea, acneiform rash, stomatitis and/or mucositis, and paronychia. In a pooled analysis of these trials (Yang, et al., 2013), grade 3 or greater toxicity occurred most commonly for diarrhea (10.3%) and rash/acne (15.4%) and led to dose reductions in 13.7% and 16.7% of patients, respectively. Other grade 3 or greater adverse events included mucositis/stomatitis (7.1%), paronychia (5.6%), decreased appetite (3.4%), vomiting (2.8%), and fatigue (2.6%). In the LUX-Lung 6 trial (Wu et al., 2014), alanine transaminase levels were found to be elevated in 20.1% of patients, 1.7% of which were found to be grade 3 or greater. Rare but serious toxicity reported across clinical studies included ocular toxicity (primarily keratitis), cardiovascular toxicity (changes in left ventricular ejection fraction), and pulmonary toxicity (often manifesting as interstitial lung disease; Katakami et al., 2013; Sequist et al., 2013; Wu et al., 2014; Miller et al., 2012).

## Role of Afatinib in NSCLC Treatment

Afatinib has demonstrated improved PFS and ORR compared with chemotherapy in *EGFR* mutation–positive patients in two phase III trials (Sequist et al., 2013; Wu et al., 2014). As a result, it is currently approved by the FDA for treatment as first-line therapy in patients with metastatic NSCLC with exon 19 deletions or exon 21 (*L858R*) substitution mutations (Boehringer Ingelheim Pharmaceuticals, 2015a). Without direct comparative trials, it is difficult to assess whether afatinib is the superior agent for those with *EGFR* mutation–positive disease, as erlotinib and gefitinib have also shown superior PFS and/or ORR in the first-line setting vs. chemotherapy (Zhou et al., 2011; Rosell et al., 2012; Mok et al., 2009; Mitsudomi et al., 2010; Maemondo et al., 2010). Afatinib’s adverse event profile appears to be similar to that seen with other EGFR tyrosine kinase inhibitors, although the rates of diarrhea and stomatitis seemed more prevalent in the LUX-Lung 3 and LUX-Lung 6 trials than those seen with erlotinib (Yang et al., 2013; Zhou et al., 2011; Rosell et al., 2012).

Afatinib may have efficacy in patients previously treated with EGFR tyrosine kinase inhibitors, as shown in the LUX-Lung 1 and LUX-Lung 4 trials. In LUX-Lung 1, a PFS advantage was found for afatinib vs. placebo, although it did not result in an OS advantage (Miller et al., 2012). However, patients considered to have acquired resistance to previous EGFR tyrosine kinase inhibitors also had a PFS of 4.5 months, similar to that found in the phase II LUX-Lung 4 trial. These data suggest that afatinib may have a benefit in resistant patients.

Overall, afatinib is a reasonable option for first-line therapy in patients with *EGFR* mutation–positive metastatic NSCLC, as it has demonstrated improved efficacy when compared with chemotherapy. Afatinib is currently recognized by the National Comprehensive Cancer Network (NCCN) as a category 1 first-line option for *EGFR*-mutated NSCLC (Ettinger et al., 2014). Further studies will be needed to determine whether afatinib is the preferred strategy when compared with first-generation agents. This strategy may be particularly true for those with exon 19 deletions. Additionally, data from the LUX-Lung 8 trial suggest that afatinib is superior to erlotinib in the second-line setting for those with squamous cell histology following initial platinum-based therapy.

## Implications for the Advanced Practitioner

Afatinib is a convenient FDA-approved option for patients with advanced NSCLC who are harboring *EGFR* exon 19 deletions or exon 21 (*L858R*) substitution mutations. It has shown an advantage compared with chemotherapy in the first-line setting for patients with *EGFR*-positive NSCLC.

However, afatinib’s role as the preferred agent in the first-line setting is debatable, although many practitioners will choose to use it due to its efficacy and convenience. Therefore, it is important for advanced practitioners to understand afatinib’s dosing, monitoring, and adverse-event profile when treating patients with advanced NSCLC. The recommended dose of afatinib is 40 mg by mouth once daily until disease progression and/or toxicity not tolerated by the patient (Boehringer Ingelheim Pharmaceuticals, 2015a).

Exposure to afatinib is significantly reduced when taken with a high-fat meal (Yap et al., 2010). Therefore, it is recommended to take afatinib on an empty stomach. Patients should be counseled not to eat at least 1 hour before and for at least 2 hours after taking afatinib. There are no defined dose reductions for renal or hepatic impairment; however, it is recommended to hold therapy if grade 2 or greater renal impairment or worsening liver function occurs during treatment with afatinib (Boehringer Ingelheim Pharmaceuticals, 2015a).

Adverse events to afatinib appear to be similar to those reported with erlotinib and gefitinib, with gastrointestinal and cutaneous effects being the most common. An acne-like rash can often be bothersome to patients and has the potential to become a serious cutaneous toxicity if not managed appropriately. Patients should be evaluated for and instructed to report skin toxicity to determine whether management with agents such as topical corticosteroids and/or topical or systemic antibiotics is warranted, according to current guidelines (Lacouture et al., 2011). It is also recommended to hold therapy for any grade 2 cutaneous reactions that have lasted for more than 7 days or are intolerable (Boehringer Ingelheim Pharmaceuticals, 2015a).

Rates of diarrhea and stomatitis seem particularly high with afatinib, and patients should be monitored closely for these adverse effects to modify dosing or discontinue treatment if warranted. Although discontinuation rates in phase III trials were less than 1% for these effects, it is recommended to withhold afatinib for grade 2 or higher diarrhea persisting for 2 or more consecutive days while taking antidiarrheal medication (Yang et al., 2013; Boehringer Ingelheim Pharmaceuticals, Inc., 2015a).

Rare ocular reactions, primarily keratitis, have been reported in limited numbers of patients using afatinib; therefore, patients should be monitored for symptoms such as eye inflammation, eye pain, or blurry vision. Changes in respiratory function should also be monitored for signs or symptoms of interstitial lung disease. Due to increased liver enzymes in some trials, liver function should be evaluated periodically and/or as clinically indicated.

If any grade 3 or higher drug-related adverse events occur while a patient is receiving afatinib therapy (or grade 2 diarrhea or cutaneous reactions as described previously), it is recommended to hold therapy until the reaction fully resolves, improves to grade 1, or returns to baseline. When resuming therapy, a reduced dose of 10 mg/day less than the previous dose is recommended (Boehringer Ingelheim Pharmaceuticals, 2015a).

Afatinib is a substrate for and inhibitor of P-glycoprotein (P-gp). It is recommended to increase the dose of afatinib by 10 mg as tolerated if using concomitant P-gp inducers such as phenytoin, phenobarbital, carbamazepine, or St. John’s wort. It is also recommended to decrease the dose by 10 mg as tolerated when using concomitant P-gp inhibitors such as ritonavir, cyclosporine A, ketoconazole, or verapamil (Boehringer Ingelheim Pharmaceuticals, 2015a).

The cost of afatinib therapy can be considerable ($7,768 average wholesale price per month’s supply [Truven Health Analytics, 2015]), and assistance programs are available to those who qualify for the program. Additionally, access to afatinib may be limited to select specialty pharmacies (see Solutions Plus, Boehringer Ingelheim Pharmaceuticals, 2015b).

## Conclusion

Treatment of advanced NSCLC is rapidly evolving, and patients with tumors that have molecular biomarkers have increased therapeutic options. Afatinib is an orally available agent with increased efficacy compared with chemotherapy, making it an attractive option for advanced NSCLC in those with adenocarcinoma and common *EGFR* mutations (Sequist et al., 2013; Wu et al., 2014). Some data suggest that afatinib can also improve outcomes in patients with resistance to other EGFR tyrosine kinase inhibitors and/or patients with squamous cell carcinoma in the second-line setting. Further studies are warranted to confirm afatinib’s place in these patient populations. However, due to the continued use of afatinib in *EGFR* mutation–positive patients with advanced NSCLC, the advanced practitioner will need to be properly trained to educate, prescribe, and monitor patients receiving it.
